# Identification Method for Internal Forces of Segmental Tunnel Linings via the Combination of Laser Scanning and Hybrid Structural Analysis

**DOI:** 10.3390/s22062421

**Published:** 2022-03-21

**Authors:** Yumeng Zhang, Jurij Karlovšek, Xian Liu

**Affiliations:** 1 College of Civil Engineering, Tongji University, Shanghai 200092, China; zhangym@tongji.edu.cn; 2 School of Civil Engineering, The University of Queensland, Brisbane, QLD 4072, Australia; j.karlovsek@uq.edu.au; 3 State Key Laboratory for Hazard Reduction in Civil Engineering, Tongji University, Shanghai 200092, China

**Keywords:** segmental lining, hybrid analysis, first-order arch theory, bending moments

## Abstract

This paper provides a new solution to identify the internal forces of segmental tunnel linings by combining laser scanning and hybrid structural analysis. First, a hybrid structural analysis method for quantifying the internal forces based on displacement monitoring is established, which requires comprehensive displacement monitoring with high precision and a complete trace history. Motivated by the development of laser scanning, two remedial solutions are proposed for typically insufficient engineering conditions, i.e., lack of displacement developing process and poor accuracy of measurements, which is highlighted in this paper. Therefore, with the help of remedial solutions, the structural analysis is able to be adopted with the application of laser scanning. The tool for developing remedial solutions is the first-order theory of slender circular arches. Virtual tests, based on a calibrated finite element model, were performed to verify the feasibility of the presented hybrid analysis and remedial solutions. In addition, parametric analyses were conducted to study the error propagation from laser scanning to the results of hybrid analysis. The resolution and measurement noise of laser scanning were investigated and discussed. On this basis, advice on combining laser scanning and hybrid structural analysis is proposed. Finally, on-site application of the hybrid analysis on an actual tunnel is presented and discussed.

## 1. Introduction

With high mechanical efficiency and great environmental friendliness, segmental linings have been widely used in the construction of tunnels in urban areas. These linings, which may serve for transportation, water conveyance, or pipelines, are usually designed to work for decades or even more than 100 years. However, with mechanical and physical attacks happening occasionally, the structure’s decays are unavoidable, which raises concerns about the safety of the segmental linings [1]. This calls for a methodology to quantify the internal forces of segmental linings, since the behavior of structures is governed by the distribution of internal forces.

Normally, quantifying the internal forces of a structure requires an awareness of the loading scenario, including the distribution and magnitude. Although some design codes [2,3] provide the methodology to estimate the loading scenario, the real load borne by the structure is still unobtainable. Because the loads estimated according to the design code are regressed from vast empirical practices, it is for covering most structural design cases but not for a particular case of structural assessment [4]. Thus, for structural assessment, it is not suitable to quantify the internal forces directly according to the loads suggested in the design code. Compared with aboveground buildings, the actual loads of underground structures are even more difficult to obtain because the water and soil pressure are extremely challenging to predict [5]. Some on-site sampling tests have been employed to determine the load on the drilling points [6]. However, due to the inherent high uncertainty of the stratum, the loading distribution is still not available from limited sampling tests. Therefore, the calculated internal forces often differ significantly from the real values as the input loads are not accurate.

To evade the problem in quantifying the loading scenario, there are methods to quantify the internal force using data coming from on-site displacement monitoring. Hellmich et al. [7] came up with a hybrid method for quantifying the stress state in shotcrete tunnel shells in the framework of a non-linear finite element method, where the input is the in-situ 3D displacement history of tunnel linings. Fuentes [8] presented an analytical solution calculating the bending moments of piles, retaining walls based on the principle of virtual work. The input of the solution comes from on-site measured displacement perpendicular to the longitudinal axis. The results of the solution were checked by comparison with those of the published literature. He et al. [9] proposed a back-analysis method to analyze the internal forces of primary support in deep tunnels based on the theory of a circular curved beam, where the input of is the in-situ-measured radial displacement of primary linings and contact stress between shotcrete and surrounding rocks.

The above-mentioned methods regard the tunnel lining as totally homogenous and continuous, which is not true for segmental tunnel linings, because the deformation configuration of segmental tunnel linings is not continuous but depends on the interfacial behavior of the longitudinal joints. Lackner et al. [10] extended the hybrid method (proposed by Hellmich et al. [7]) to the analysis of segmented shotcrete tunnel linings, in which the displacement field is assumed to be smooth within each segment but not the whole lining. Zhang et al. [11,12] and Jiang et al. [13] proposed a hybrid method to analyze the segmental tunnel linings, where the dislocation of longitudinal was regarded as one of the input parameters, avoiding the complex work to simulate the joint behavior. These two hybrid methods required the use of numerous embedded displacement sensors on the joints of the lining from the very beginning of tunnel construction, which is not economical.

Laser scanning is a new method employed in tunnel routine inspection, able to acquire the internal profile of a tunnel in a relatively short period [14], inspiring the authors to build a hybrid analysis method to quantify the internal forces of segmental tunnel linings using the data acquired from laser scanning. However, there are two factors hindering the combination of laser scanning and hybrid structural analysis. One refers to the low measurement accuracy: in recent years, although the measurement resolution of laser scanners has increased significantly, it does not meet the high precision requirement of traditional hybrid structural analysis. The other one refers to the lack of the developing process of displacement: the laser scanning only provides the profile of deformed linings, without considering the developing process of the deformed configuration of the lining. Regarding all the above, this paper provides remedial solutions regarding the combination of laser scanning and hybrid structural analysis.

In Section 2, a displacement-based hybrid structural analysis method for quantifying the internal forces based on displacement monitoring is established. Then, in Section 3, considering the practical process of laser scanning, remedial solutions are proposed for typically insufficient conditions, i.e., lack of displacement history and poor accuracy of measurements. In Section 4, virtual tests, based on a calibrated finite element model, are performed to verify the feasibility of the presented hybrid analysis, and remedial solution I and II. In Section 5, parametric analyses are conducted to study the error propagation from laser scanning to the results of hybrid analysis. The resolution and measurement noise of laser scanning are investigated and discussed. On this basis, advice on combining laser scanning and hybrid structural analysis is proposed. In Section 6, on-site application of the hybrid method on an actual metro tunnel is presented and discussed. Conclusions are given in Section 7. Moreover, Appendix A illustrates the rationality of Equation (7), which is a further explanation for remedial solution II. Appendix B contains a list of symbols.

## 2. Hybrid Structural Analysis

This section presents a hybrid method to analyze the circular segmental tunnel linings, where the word ‘hybrid’ represents the combination of measurement data with advanced simulation tools of engineering mechanics [15]. The circular segmental tunnel linings will be referred to as the ‘segmental lining ring’ for simplicity in the following text. The hybrid method was first introduced in a new-Austrian-tunnelling-method tunnel [7], based on which the authors extended it to segmental tunnel linings. The input of the hybrid analysis is the displacement of measurement points on the segment body, and the output is the internal force of the segmental lining ring.

### 2.1. Assumptions

The assumptions employed for the hybrid structural analysis are listed below.

A1: The strain of the segment is caused by stress in a relatively short period. By this assumption, the conditions for the hybrid analysis are limited to newly built tunnels. The deformation caused by creep and environment is ignored. In addition, this means that the segments are free of stress at their initial state where they are undeformed.

A2: The displacement of the segment is small compared with the cross-sectional dimension. This assumption is made for the fact that the basis of this hybrid analysis is the first-order theory of a circular slender arch; thus, the assumption of the first-order theory of a circular slender arch should also be followed.

### 2.2. Procedure for Hybrid Structural Analysis

The procedure for hybrid structural analysis is introduced step by step.

S1: Reconstruct the displacement field of the segmental lining ring.

**Figure 1:**
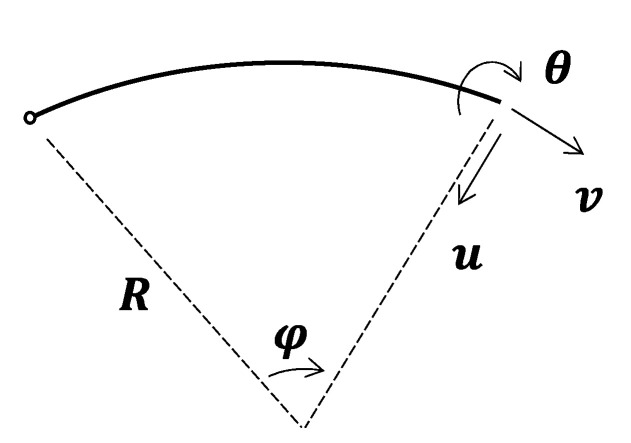
The direction of displacement.

The displacement configuration of each segment forms a smooth curve, which has been illustrated in the authors’ previous research [16]. Hence, the displacement of each segment can be interpolated by a few measurement points. Trigonometric polynomials (see Equations (1) and (2)) are recommended by the authors, with which both the radial and tangential displacement can be well approximated with a low risk of over-fitting:

(1) $$u\left(\varphi \right)=C_{1}\varphi \cos\left(\varphi \right)+C_{2}\varphi \sin\left(\varphi \right)+C_{3}\cos\left(\varphi \right)+C_{4}\sin\left(\varphi \right)+C_{5},$$

(2) $$v\left(\varphi \right)={C^{\prime }}_{1}\varphi \cos\left(\varphi \right)+{C^{\prime }}_{2}\varphi \sin\left(\varphi \right)+{C^{\prime }}_{3}\cos\left(\varphi \right)+{C^{\prime }}_{4}\sin\left(\varphi \right)+{C^{\prime }}_{5},$$

where u represents the radial displacement; v denotes the tangential displacement; the φ symbolizes the polar angle coordinate of the segment node. The direction of displacement is as defined in Figure 1. $C_{1}\sim C_{5}$ and ${C^{\prime }}_{1}\sim {C^{\prime }}_{5}$ stand for the constants, which can be solved by k measurement points (k ≥ 5):

(3) $$\left[\begin{array}{c} u_{1}\\ u_{2}\\ \vdots \\ u_{k} \end{array}\right]=\left[\begin{array}{ccccc} \varphi_{1}\cos\left(\varphi_{1}\right) & \varphi_{1}\sin\left(\varphi_{1}\right) & \cos\left(\varphi_{1}\right) & \sin\left(\varphi_{1}\right) & 1\\ \varphi_{2}\cos\left(\varphi_{2}\right) & \varphi_{2}\sin\left(\varphi_{2}\right) & \cos\left(\varphi_{2}\right) & \sin\left(\varphi_{2}\right) & 1\\ \vdots & \vdots & \vdots & \vdots & \vdots \\ \varphi_{k}\cos\left(\varphi_{k}\right) & \varphi_{k}\sin\left(\varphi_{k}\right) & \cos\left(\varphi_{k}\right) & \sin\left(\varphi_{k}\right) & 1 \end{array}\right]\left[\begin{array}{c} C_{1}\\ C_{2}\\ C_{3}\\ C_{4}\\ C_{5} \end{array}\right],$$

where $\varphi_{1}$~$\varphi_{k}$ stand for the polar coordinate of measurement points 1~k, and $u_{1}$~$u_{k}$ denote the radial displacement of measurement points 1~k. If k=5, the equation has a unique solution; if k>5, the equation can be solved by optimization, such as the least squares method. The same approach can be adopted to determine the constants ${C^{\prime }}_{1}\sim {C^{\prime }}_{5}$.

In addition, the field of rotation angles are also required for the hybrid analysis. In the event, that inclinometers are not arranged, the rotation angle can be estimated by the following equation:

(4) $$\theta \left(\varphi \right)=\frac{1}{R}\frac{du\left(\varphi \right)}{d\varphi }+\frac{v\left(\varphi \right)}{R},$$

where *R* is the average radius of the lining ring. Equation (4) is extracted from the first-order theory of the slender arch [17].

S2: Set up a finite element model of the segmental lining ring.

The FEM model should be established according to the initial geometry of the segmental lining ring. Only the modeling of the segment is required and there is no need to model the joint or geological stratum, because the translation and rotation of segment nodes have provided enough constraints to solve the internal forces. Considering the segment to be a curve beam, every element node has three degrees of freedom, including two for translation and one for rotation, as is shown in Equation (5).

(5) $$u_{e}={\left(u_{ei},v_{ei},\theta_{ei}\right)}^{T},$$

where $u_{e}$ is the displacement vector of each element node, and subscript i denotes the node number.

S3: Determine the displacement of element nodes.

Assume that the polar coordinate of element nodes is $\varphi_{1}^{e}$, $\varphi_{2}^{e}$,…$\varphi_{m}^{e}$, where the superscript e means the element node and subscript 1, 2,…m represent the number of each element node. Then, the displacement component of each element node can be determined by the equations in Step 1:

(6) $$u_{e}={\left(u_{ei},v_{ei},\theta_{ei}\right)}^{T}={\left[u\left(\varphi_{i}^{e}\right),v\left(\varphi_{i}^{e}\right),\theta \left(\varphi_{i}^{e}\right)\right]}^{T},$$

S4: Quantify the internal forces of the simulated tunnel linings.

Displacement-controlled FEM is employed for quantifying the internal forces of the segmental tunnel rings [18,19]. Contrary to a force-controlled analysis, displacement-controlled analysis changes the displacement incrementally, while the reaction forces depend on the stiffness of the element. In this step, the tangential displacement, radial displacement, and rotation of each node in Step 3 are constrained incrementally until the displacement in Step 3 is reached. Consequently, the strain field of segmental linings is determined by the displacement field deduced in Step 3, and the stress field is further determined by the constitutive model of segment materials.

For now, a hybrid structural analysis for quantifying the internal forces based on displacement monitoring has been established. Henceforth, in the paper, the method presented in this section will be referred to as the ‘original hybrid analysis’ for simplicity. It is worth noting that the original hybrid structural analysis is difficult to be adopted on-site because it requires comprehensive displacement monitoring with high precision and complete trace history. To make the hybrid structural analysis more practical, the combination of laser scanning and hybrid structural analysis is discussed in the following section.

## 3. Combination of Laser Scanning and Hybrid Structural Analysis

### 3.1. Data Acquisition of Laser Scanning

Laser scanning is a fast-developing method for identifying the profile of tunnels, which is known for its labor savings and time savings. It employs a laser emitter to generate a laser plane, which intersects a bright tunnel profile on the inner surface of the tunnel [14]. The laser beam travels from the laser emitter and bounces off tunnel surfaces, reflecting back to the emitter. By measuring the time journey of the laser beam, the distance between the laser emitter and the tunnel surface is identified. Via data processing, the profile of the tunnel lining is described as a series of coordinate points. The number of points obtained per scan ranges from hundreds to thousands, depending on the time of the scanning. The number of measurement points provided by laser scanning is enough for hybrid structural analysis. However, there are two weaknesses that hinder the combination of laser scanning and hybrid structural analysis.

(1) Lack of displacement history

Although the profile of the tunnel lining is described as a series of plane coordinate points, these represent the profile of the currently deformed lining and contain no information of the displacement history. To reconstruct the displacement field of the segmental lining, the initial position of each scanned cloud point is required. To solve the problem of lacking displacement history, the authors propose a remedial solution backtracking the displacement of each scanned cloud point, which will be presented in Section 3.2.

(2) High precision requirement on displacement measurement

Due to the high stiffness of concrete components, displacement-based solutions usually have a high precision requirement on displacement measurement. From the authors’ numerical study, the reasonable results of original hybrid analysis only occur at the condition that the measurement precision is within 0.01 mm, which is extremely difficult to achieve for laser scanning. Regarding this, the authors propose a remedial solution lowering the requirement of the measurement precision of using hybrid structural analysis, which will be discussed in Section 3.3.

### 3.2. Remedial Solution I—Lack of Displacement History

#### 3.2.1. Philosophy of Remedial Solution I

As prefabricated components, segments are designed and manufactured in the philosophy of modular production, resulting in relatively high manufacture quality compared to the on-site casting components. Thus, it is simple to track the initial profile of the segment. By comparing the initial profile and current profile of the segment, the initial position of each measurement point can be inferred. Then, the displacement of each measurement point can be regarded as the vector from the initial position to the current position.

#### 3.2.2. Additional Assumptions

An additional assumption is introduced for the solution.

A3: The segment is well manufactured as a circular arc, and the segmental lining ring is well assembled. Taking the Shanghai Metro tunnel as an example, it has an outer diameter of 6.2 m and an inner diameter of 5.5 m. The thickness of each segment is 0.35 m. Every segmental ring contains 6 segments including a key segment. In addition, every segment is connected by bolts to ensure integrity. It is stipulated by the code CJJ/T 164-2011 [20] that the profile of the segment should be checked before leaving the factory. The assembled segmental ring in Shanghai is required to approximate a circular arc with an error of less than 2 mm according to the code. Thus, the assumption is reasonable.

#### 3.2.3. Procedure for Remedial Solution I

The input of remedial solution I is the coordinate $\left(r_{i},\varphi_{i}\right)$ of the scanned points, where $r_{i}$ is the radial coordinate of scanned point i and $\varphi_{i}$ is the polar coordinate of this. By remedial solution I, the corresponding displacement $\left(u_{i},v_{i}\right)$ of each scanned point can be backtracked.

RS1: Approximate the configuration of every segment r=r(φ) based on the coordinates of scanned point $\left(r_{i},\varphi_{i}\right)$. The function of Equation (7) is recommended by the authors to conduct the approximation:

(7) $$r\left(\varphi \right)={C^{\prime \prime }}_{1}\varphi \cos\left(\varphi \right)+{C^{\prime \prime }}_{2}\varphi \sin\left(\varphi \right)+{C^{\prime \prime }}_{3}\cos\left(\varphi \right)+{C^{\prime \prime }}_{4}\sin\left(\varphi \right)+{C^{\prime \prime }}_{5},$$

At least 5 measurement points are required for solving the undetermined constants ${C^{\prime \prime }}_{1}\sim {C^{\prime \prime }}_{5}$.

**Figure 2:**
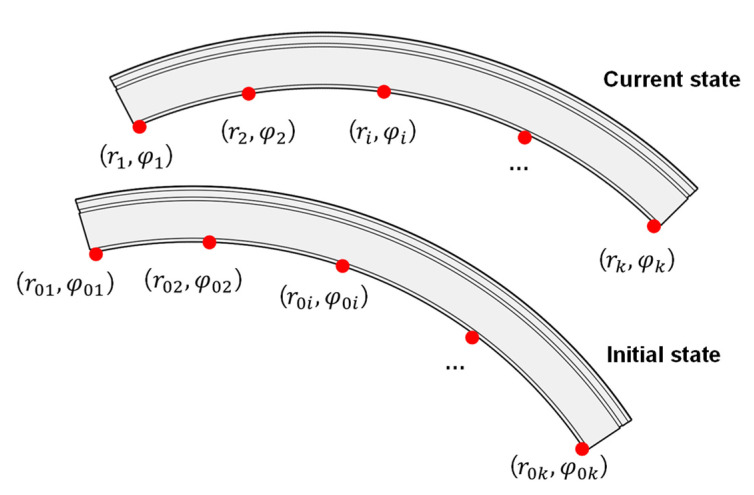
The schematic diagram of initial position and current position of segment.

RS2: Determine the initial position $\left(r_{0i},\varphi_{0i}\right)$ of scanned points. Here, $r_{0i}$ is the initial radial coordinate of scanned point i, and $\varphi_{0}$ is the initial polar coordinate of this (see Figure 2). By the assumption of A2, the initial radial coordinate of all scanned points can be set as designed radius R. The initial polar coordinate of scanned point i is denoted by Equation (8). The derivation of the equation is based on the discovery that the arc length between each measurement point is proportional during the process of deformation, which is explained in detail in Appendix A.

(8) $$\varphi_{0i}=\frac{\int_{\varphi_{1}}^{\varphi_{i}}\sqrt{r\left(\varphi \right)+r^{\prime }\left(\varphi \right)}d\varphi }{\int_{\varphi_{1}}^{\varphi_{k}}\sqrt{r\left(\varphi \right)+r^{\prime }\left(\varphi \right)}d\varphi }\left(\varphi_{0k}-\varphi_{01}\right)+\varphi_{01},$$

The $r^{\prime }\left(\varphi \right)$ is the first derivation of r(φ) in RS1. Note that $\varphi_{01}$ and $\varphi_{0k}$ represent the initial polar coordinate of both ends of the segment, based on the additional assumption A2; $\varphi_{0k}$ and $\varphi_{01}$ can be attained from the designed configuration. $\varphi_{1}$, $\varphi_{i}$, and $\varphi_{k}$ are obtained from laser scanning, where $\varphi_{1}$ and $\varphi_{k}$ represent the measured polar coordinate of both ends of segment. Thus, $\varphi_{0i}$ is solved and the initial coordinate of the scanned point i is determined as (R,$\;\varphi_{0i}$).

RS3: Determine the displacement of each scanned point. The displacement of each scanned point i can be exported by subtracting the initial coordinate from the current coordinate according to Equations (9) and (10):

(9) $$u_{i}=r_{i}-R,$$

(10) $$v_{i}=R\cdot \left(\varphi_{i}-\varphi_{0i}\right),$$

### 3.3. Remedial Solution II—Poor Precision of Measurements

#### 3.3.1. Philosophy of Remedial Solution II

To evade the high precision requirement of the original hybrid method, two measures are conducted.

On the one hand, the hybrid structural analysis relies on the solution of the displacement-controlled FEM method, which is naturally sensitive to the error of displacement input. Compared to bending moments, the axial forces rely much more on the high accuracy of measurement because the compression stiffness EA of the segment is more than 100 times larger than the bending stiffness EI for a typical configuration of the metro tunnel. This indicates that the analysis of bending moments from displacement is more economical than that of axial forces. Furthermore, the axial force of the segmental lining ring in the soft soil region is relatively easy to calculate from the vertical load, and the vertical load can be estimated by simply imposing the gravity of the stratum. Based on all the above, the authors decided to quantify the axial force based on the vertical load rather than on the displacement.

On the other hand, the radial displacement of the segmental lining ring is larger than the tangential displacement in most sections, which will be discussed in Section 4.3. Given the same measurement method, the relative error of radial displacement will be less than that of tangential displacement. Thus, the author chose to regard the tangential displacement as a variable with high uncertainty. In remedial solution II, the tangential displacement will be derived from radial displacement rather than from the results of on-site tangential displacement monitoring, improving the reliability of the tangential displacement input.

#### 3.3.2. Additional Assumptions

Two additional assumptions are introduced for the solution.

A4: The axial force is a constant within each segment. The assumption is made since the distribution of axial forces of most segmental linings is uniform, which is especially true for the linings buried at a large depth [21,22].

A5: The material of segments is elastic. Although the nonlinear behavior is ignored, the solution in this section is still prospective because the deformation of segmental linings is mainly caused by the rotation and detachment of the longitudinal joint, while, in most cases, the segment bodies are slightly deformed and remain elastic.

#### 3.3.3. Procedure for Remedial Solution II

The input of remedial solution II consists of the displacement of each measurement point $\left(u_{i},v_{i}\right)$ and the estimated vertical load above the tunnel crown. The output is the displacement field of the segment.

RS1’: Reconstruct the radial displacement field u(φ) of the segment, following Step S1 of Section 2.2.

RS2’: Estimate the axial force of the segment. The axial force N can be estimated by Equation (11), which is simply imposing the gravity of the stratum:

(11) $$N=\frac{1}{2}\sum \gamma_{i}H_{i}Rb,$$

where $\gamma_{i}$ and $H_{i}$ are the gravity density and height of the stratum over the crown of the tunnel lining. R denotes the average radius of segmental tunnel linings, and b represents the width of the segment. $\sum^{}\gamma_{i}H_{i}Rb$ can be replaced by an estimated vertical load of the segmental lining ring.

RS3’: Calculate the tangential displacement field v(φ). The following equation is employed to estimate the tangential displacement, which is extracted based on the theory of the elastic slender arch (Dym et al., 2011):

(12) $$v\left(\varphi \right)=\int^{}\left(\frac{NR}{EA}-u\left(\varphi \right)\right)d\varphi +X_{1}\cos\left(\varphi \right)+X_{2}\sin\left(\varphi \right)+X_{3},$$

where u(φ) is the radial displacement field derived in RS1’; $X_{1}\sim X_{3}$ are undetermined constants. Thus, v(φ) can be solved by at least 3 measurement points.

RS4’: Calculate the rotation angle field θ(φ) according to Equation (4).

## 4. Numerical Validation

### 4.1. A Virtual FEM Test

**Figure 3:**
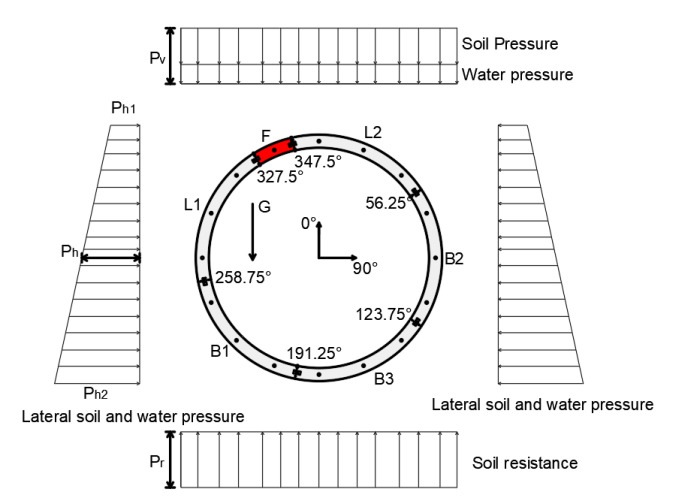
The exemplary assembly and load distribution of the segmental tunnel ring.

The purpose of the virtual test is to obtain accurate data for evaluating the original hybrid structural analysis method and its remedial solutions. The virtual test is conducted based on a nonlinear FEM model, which has been introduced and validated in the authors’ previous work [16]. Typical segmental lining rings of the railway metro system are employed in the virtual test. For the sake of simplicity, only one ling ring was modeled; see Figure 3. To ensure the universality of the virtual test, the lining is arranged to be asymmetrical about 0–180°, where 0° indicates the crown of the lining and 180° represents the floor. The lining ring has an outer diameter of 6.6 m and inner diameter of 5.9 m. The lining ring consists of six segments including a key segment, shown as the red part with capital F in Figure 3. The segments are made of reinforced concrete with a thickness of 0.35 m, and the ring width is 1.2 m.

In the virtual FEM test, the lining ring was subjected to water and soil pressure. The load distribution of the lining ring is shown in Figure 3, which is suggested by the design guidelines of the International Tunnel Association [2]. The load parameters, corresponding to a real tunnel buried at a depth of 10 m, are shown in Table 1. They are calculated by the stratum parameters of a real tunnel in Ningbo, a soft soil area in China. The FEM test was implemented in the commercial software LS-DYNA^®^. For the sake of brevity, the results of the FEM test will be introduced together with the original hybrid analysis in Section 4.3.

### 4.2. Design of Validation

**Figure 4:**
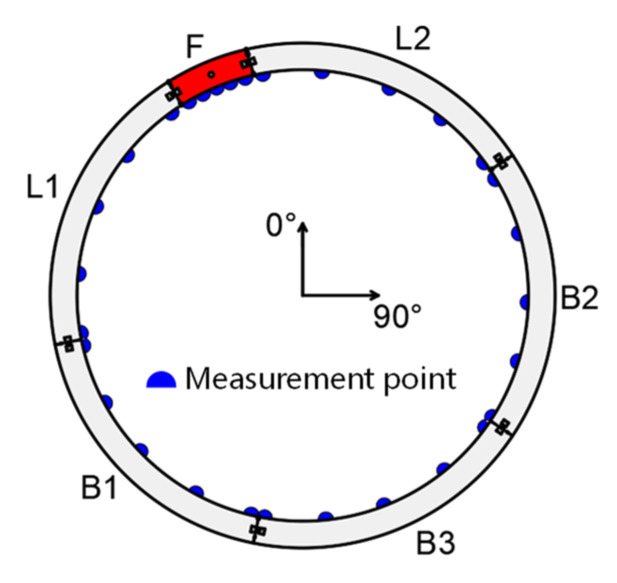
The position of virtual measurement points.

After running the model, the internal forces, i.e., bending moments and axial forces, of each element were recorded, which would be the baseline for validating the proposed method in the paper. Five virtual measurement points for displacement were arranged on each segment (see Figure 4). The radial displacement $u_{i}$, tangential displacement $v_{i}$, radial coordinate $r_{i}$, and polar coordinate $\varphi_{i}$ of each measurement point were extracted from the results of the FEM model. These displacements or coordinates would be employed as input for the original hybrid solution, as well as remedial solution I and II.

Four groups of hybrid analyses are arranged for validation, see Table 2. For Group 1, the displacements of measurement points ($u_{i},v_{i})$ were employed as the input of the original hybrid analysis, following the steps in Section 2.2. Group 2 combined the hybrid analysis with remedial solution I and employed the coordinates of measurement points ($r_{i},\varphi_{i}$) as input instead of $\left(u_{i},v_{i}\right)$. It followed the sequence RS1→RS2→RS3→S1→S2→S3→S4. As for Group 3, a poor measurement condition was simulated by manually giving the displacement of each measurement point with an evenly distributed error of 0.1 mm. Thus, the input of Group 3 was errored displacement $\left({u^{\prime }}_{i},{v^{\prime }}_{i}\right)$ and estimated vertical load Pv. Group 3 was conducted following the sequence RS1’→RS2’→RS3’→RS4’→S2→S3→S4. To enrich the results, Group 4 was arranged where the original hybrid analysis with errored displacement $\left({u^{\prime }}_{i},{v^{\prime }}_{i}\right)$ was conducted. In the following sections, the displacements and internal forces of each group are extracted and compared with the results of the virtual FEM test, therefore evaluating the proposed original hybrid analysis and remedial solution I and II.

### 4.3. Validation of Original Hybrid Analysis

**Figure 5:**
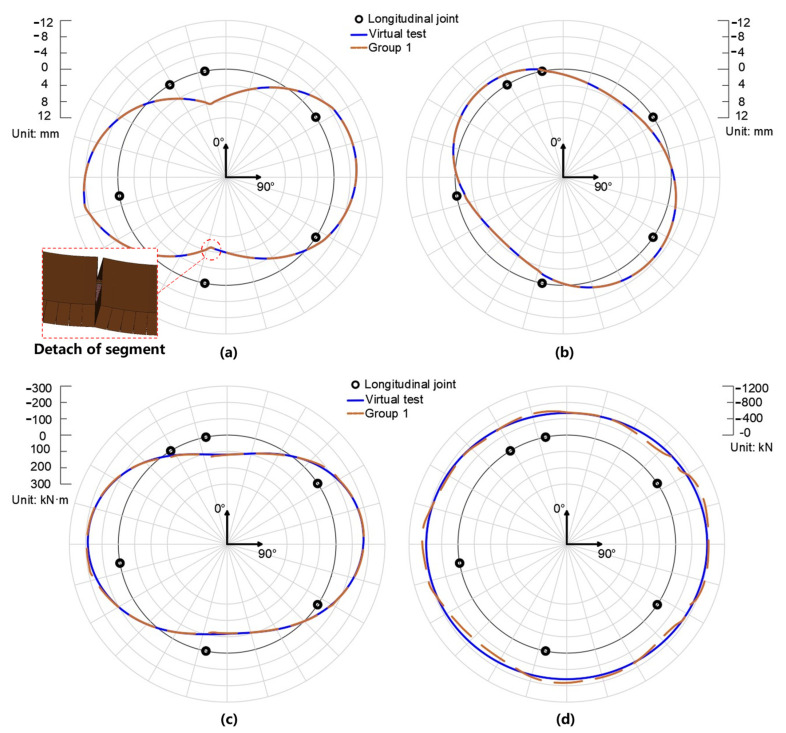
The radial displacement (**a**), tangential displacement (**b**), bending moment (**c**), and axial force (**d**) of virtual test and Group 1.

Figure 5a,b show the radial and tangential displacement of the virtual test and Group 1. The former is defined as positive when the ring moves inward. The latter is defined as positive when the ring rotates clockwise. As is shown in Figure 5a, the lining ring deformed to be an oval in terms of radial displacement, moving inwards at the crown and bottom, and expanding outwards at two waists. It is noteworthy that although the radial displacement field is smooth within every segment, the tendency of radial displacement at one side of every longitudinal joint does not always match that at the other side. This is because of the detachment of the neighboring segments (see Figure 5a). As for the tangential displacement, the upper-right and bottom-left part of the lining rotate in a clockwise direction, while the other part of the lining rotates in a counterclockwise direction (see Figure 5b). Unlike the radial displacement, the distribution of tangential displacement is influenced very slightly by the existence of the longitudinal joints. In a word, both the radial and tangential displacement of the hybrid analysis are consistent with those of the virtual test, proving the suitability of interpolation Equations (1) and (2).

Figure 5c,d show the bending moments and axial forces of the lining ring. The bending moment is defined as positive when the internal surface of the segment is in tensile. The negative axial force means compressive resultant force. In general, the bending moment is positive at the crown and floor, and gradually increases to be negative at the waist (see Figure 5c). The axial forces are visually uniformly distributed along the circumference of the lining, slightly lower at the bottom and crown than those at the waist (see Figure 5d). Furthermore, both the bending moment and axial force quantified by the original hybrid analysis are in good agreement with those of the virtual test. Hence, the rationality of the original hybrid analysis is validated.

### 4.4. Validation of Remedial Solution I

**Figure 6:**
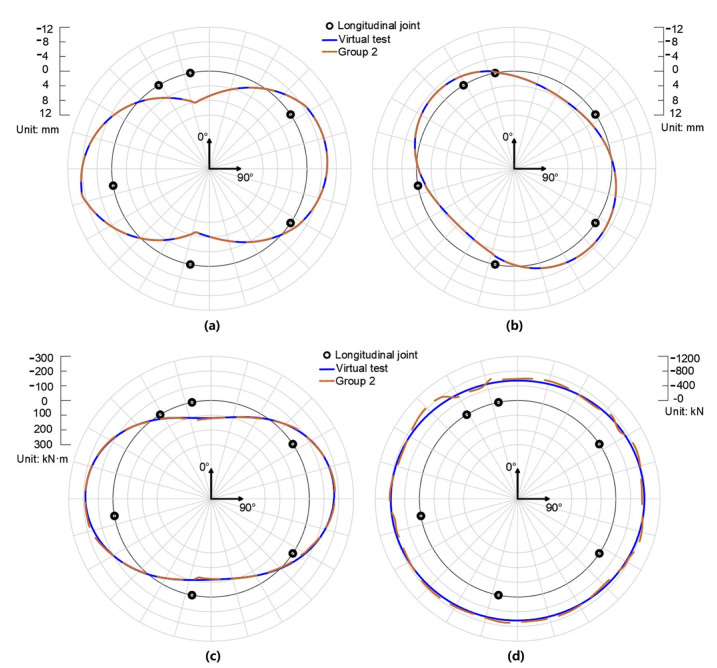
The radial displacement (**a**), tangential displacement (**b**), bending moment (**c**), and axial force (**d**) of virtual test and Group 2.

Figure 6a,b show the radial and tangential displacement of the virtual test and Group 2. The results show that although Group 2 takes the deformed profile ($r_{i},\varphi_{i}$) of measurement points as input, it reproduces the displacement field of the segmental lining ring to a great extent. The reproduced displacement can be employed for quantifying the internal forces of the segmental lining ring; see Figure 6c,d. Hence, the rationality of remedial solution I is validated.

### 4.5. Validation of Remedial Solution II

**Figure 7:**
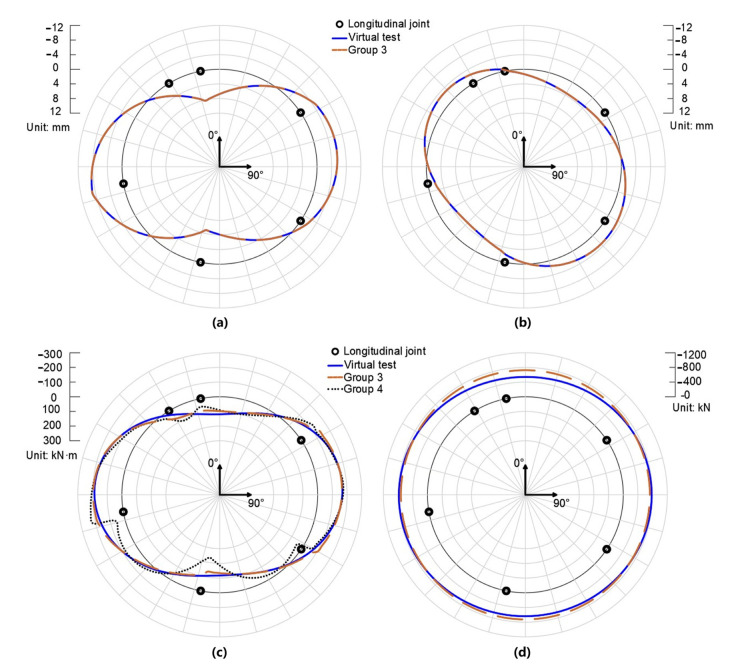
The radial displacement (**a**), tangential displacement (**b**), bending moment (**c**), and axial force (**d**) obtained by the hybrid analysis and remedial solution I at the condition that the measurement precision is 0.1 mm.

Figure 7a,b compare the radial and tangential displacement of the virtual test and Group 3. Figure 7c,d compare the bending moments and axial forces of the virtual test, Group 3, and Group 4. The bending moments of Group 4 differ from those of the virtual test, especially in the vicinity of the longitudinal joints. As for the axial force of Group 4, it was unsatisfactory, so the authors decided not to present it in the paper. This proves that the original hybrid analysis is not suitable for input with measurement error.

Meanwhile, the results of Group 3 agree well with the results of the virtual test in terms of both displacements and internal forces. It is worth noting that the bending moments of Group 3 in the vicinity of longitudinal joints show slight deviation from those of the virtual test. This is due to the discontinuity of the reconstructed displacement field since the displacement fields are reconstructed segment by segment. Thus, slight discontinuity happens given the errored displacement input. These inconsistencies of bending moments will reduce as the measurement precision increases. Apart from this, Group 3 agrees well with the results of the virtual test, indicating the rationality of remedial solution II. Compared with the results of Group 4, it is concluded that remedial solution II lowers the barrier of measurement precision effectively.

Thus far, the original hybrid analysis and remedial solution I and II have been validated numerically. With the above-mentioned solutions, the hybrid method is able to be applied to the circular segmental ring lacking the displacement history. Thus, it provides the feasibility to combine the hybrid method with laser scanning, which will be discussed in Section 5. It should be noted that the load input of the FEM test follows the loading scheme in Figure 3; thus, the validation in this section makes sense for segmental linings in soft soil areas. For tunnels with different loading conditions, the feasibility of the hybrid analysis remains to be discussed.

## 5. Parameter Analysis of Measurement Accuracy

### 5.1. Explanation of Error Propagation

The aim of this section is to discuss the error of the hybrid analysis working with laser scanning. Laser scanning describes the internal surface of segmental tunnel linings as a series of scanning point clouds $\left(r_{i},\varphi_{i}\right)$, where $r_{i}$ is the polar coordinate of scanning point i and $\varphi_{i}$ is the angular coordinate of this. Normally, a laser scanning system comprises a range measurement unit and a mechanical deflection unit [23]. The range measurement unit measures the distance from the laser emitter to the surface of the lining, producing polar coordinate $r_{i}$. The mechanical deflection unit directs the laser beam into the direction to be measured and records the azimuth, influencing angular coordinate $\varphi_{i}$.

The measurement results of laser scanning possess uncertainty for two reasons. On one hand, the indicating device of the laser scanner has a finite resolution. On the other hand, the measurement results of each unit are influenced by systematic error and random error. The systematic error is usually predictable and can be compensated or reduced by calibration, while the random error is unpredictable and cannot be eliminated totally. Thus, only the random error will be discussed in this paper. In this section, the measurement error of $r_{i}$ and $\varphi_{i}$ is set to be independent since $r_{i}$ and $\varphi_{i}$ are determined by different units of the laser scanner.

The accuracy of the hybrid analysis relies on the accuracy of measurement. Combining the hybrid analysis and the laser scanning, the procedure follows RS1→RS2→RS3→RS1’→RS2’→RS3’→RS4’→S2→S3→S4. First, the configuration of segmental linings is reconstructed from the point clouds produced by laser scanning, where errored measurement data result in an errored lining configuration. Then, the displacement field is backtracked from the reconstructed lining configuration, where the errored lining configuration causes an errored displacement field. Further, errored displacement is input in the FEM model, and consequently, errored internal forces are output.

In this section, the data of lining profile $\left(r_{i},\varphi_{i}\right)$ are obtained from the model in Section 4.1, which can be regarded as the real value. Uncertainty and error are added to $\left(r_{i},\varphi_{i}\right)$ to simulate the practical laser scanning. Due to the complicated mechanism of error propagation, the influence of the measurement error on hybrid analysis is evaluated by numerical tests. The errored lining profile $\left(r_{i},\varphi_{i}\right)$ is adopted with the hybrid method flowing the procedure RS1→RS2→RS3→RS1’→RS2’→RS3’→RS4’→S2→S3→S4. The results of internal forces are compared with their real value to evaluate the error of hybrid analysis.

### 5.2. Definition of Indices

The internal force distribution deduced from hybrid analysis is expected to approximate its real distribution. Therefore, normalized root-mean-square error (NRMSE) is employed here to evaluate the overall distribution of internal forces; see Equation (13):

(13) $$NRMSE=\frac{\sqrt{\frac{\sum_{i}^{n}{\left(X_{esi,i}-X_{real,i}\right)}^{2}}{n}}}{\bar{X_{real}}},$$

where n is the number of sections, $X_{esi,i}$ is the estimated internal force of section i, $X_{real,i}$ is the real internal force of section i, and $\bar{X_{real}}$ is the average of the real internal force. A total of four sections are selected for calculating the NRMSE, i.e., the section 0°, 90°, 180°, 270°. From the authors’ overview, when the NRMSE is less than 0.1, the results of bending moments are recognized to be acceptable. Considering the fact that the axial forces are estimated by the same equation and their distributions are relatively identical, only the results of bending moments are discussed.

### 5.3. Resolution of Laser Scanner

**Figure 8:**
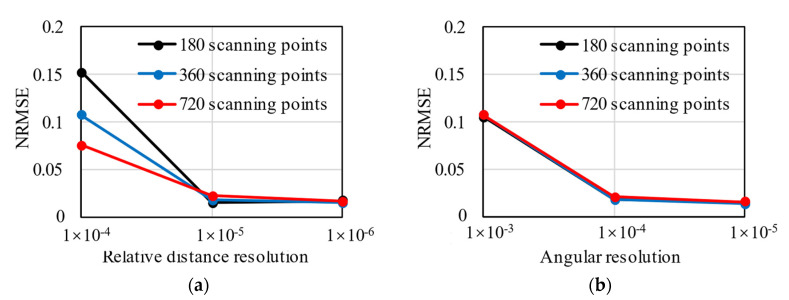
The NRMSE of hybrid analysis for various relative distance resolutions (**a**) and various angular resolutions (**b**).

A laser scanner has two independent resolutions: one refers to the distance resolution, and the other one refers to the angular resolution. For a specific distance resolution, the greater the distance from the laser emitter to the target, the less relative uncertainty produced by the distance resolution, where the relative uncertainty denotes the ratio of measurement uncertainty to the distance. Thus, to provide a reference for tunnels of different diameters, a relative distance resolution is defined here:

(14) $$Res_{r}=\frac{Res}{R},$$

where Res represents the distance resolution of the laser emitter, R denotes the radius of a circular tunnel, and $Res_{r}$ is the relative distance resolution. According to the authors’ investigation, the distance resolution of laser scanners varies from 0.1 mm to 1 cm [24,25], corresponding to the relative distance resolution of 3.3 × 10^−5^ to 3.3 × 10^−3^. In this section, numerical tests were conducted for varying relative distance resolutions and angular resolutions. The relative distance resolution starts from 1 × 10^−6^ and increases incrementally. As for the angular resolution, it starts from 1 × 10^−5^ and increases incrementally. The data of lining profile $\left(r_{i},\varphi_{i}\right)$ are obtained from the model in Section 4.1, which can be regarded as the real value. Different resolutions were simulated by rounding the $r_{i}$ and $\varphi_{i}$ with different decimal places. Scanning point clouds of three density levels are discussed, i.e., 180 scanning points per ring, 360 scanning points per ring, and 720 scanning points per ring. The results of numerical tests are shown in Figure 8.

Given the same number of scanning points, with the increase in the relative distance resolution, the NRMSE decreases. If the distance resolution of the laser scanner is δx, the real value of the measurand that produces a given indication X can lie with equal probability anywhere in the interval X−δx to X+δx [26]. Thus, the smaller the resolution, the more accurate the distance $r_{i}$, and the more reasonable the results of the hybrid analysis. When the resolution is larger than 1 × 10^−4^, the NRMSE is higher than 0.1, and the error of the hybrid analysis becomes unacceptable. The error of the hybrid analysis can be lowered by increasing the number of scanning point clouds. The more scanning points, the more likely it is that the hybrid analysis tracks the accurate displacement field; as a result, internal forces with less error are produced by the hybrid analysis. However, it should be noted that when the relative distance resolution is less than 1 × 10^−5^, NRMSE is not sensitive to the number of scanning points but remains on a low level.

As with the relative distance resolution, the NRMSE decreases with the rise of the angular resolution. When the angular resolution is less than 1 × 10^−3^, the error of the hybrid analysis is acceptable. However, NRMSE does not change visually when the number of scanning points increases. This is because the first step of hybrid analysis combined with laser scanning is to reconstruct the profile of segmental linings as r=r(φ), where φ is the independent variable, and the number of $\varphi_{i}$ does not influence the quality of profile reconstruction. Consequently, the results of hybrid analysis are not sensitive to the number of $\varphi_{i}$.

### 5.4. Noise of Measurement

**Figure 9:**
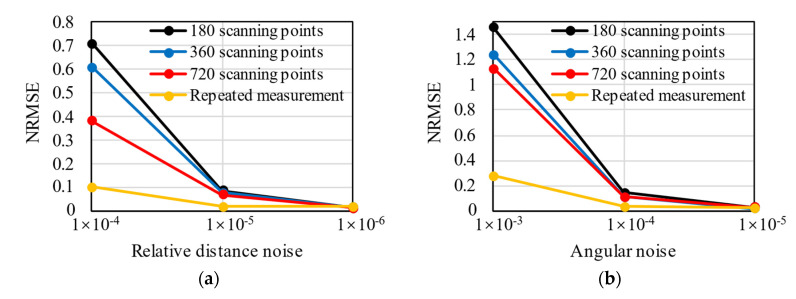
The NRMSE of hybrid analysis for various relative distance noise (**a**) and various angular noise (**b**).

Because of the random error, different indicating values can be obtained for repeated measurements. If a measurement is repeated enough times, the deviation from the indicating value to its real value follows a normal distribution $N\left(0,\sigma^{2}\right)$. The standard deviation σ is defined as the measurement noise. Moreover, the distance noise and angular noise are regarded as independent. A relative distance noise is defined here to provide a reference for tunnels of different diameters:

(15) $$\sigma_{r}=\frac{\sigma }{R},$$

where R denotes the radius of a circular tunnel, σ represents the distance noise, and $\sigma_{r}$ is the relative distance noise. In this section, numerical tests were conducted for varying relative distance noise and angular noise. The relative distance noise starts from 1 × 10^−6^ and the angular noise starts from 1 × 10^−5^. The data of lining profile $\left(r_{i},\varphi_{i}\right)$ are obtained from the model in Section 4.1, which can be regarded as the real value. A normally distributed error is superimposed to the real $r_{i}$ and $\varphi_{i}$ to simulate the measurement noise. Scanning point clouds of three density levels are discussed, i.e., 180 scanning points per ring, 360 scanning points per ring, and 720 scanning points per ring. Additionally, to enrich the results, 720 scanning points per ring with repeated measurements are discussed. This means that, for every scanning point, the measurements were conducted 20 times and the final $\left(r_{i},\varphi_{i}\right)$ is the average value of these 20-time measurements. The results of numerical tests are shown in Figure 9.

The NRMSE of hybrid analysis decreases with the reduction in the relative distance noise, and the same occurs for the angular noise. If measurements are not repeated, acceptable results only occur when the relative distance noise is less than 1 × 10^−5^ or the angular noise is less than 1 × 10^−4^. Given the same measurement noise, more scanning points help to lower the error of hybrid analysis. Repeated measurement also induces low error in the hybrid analysis. Because the measurement noise follows a normal distribution, the indicating value can lie symmetrically about the real value. If the measurements are conducted an adequate number of times, the average indicating value is expected to approximate the real value infinitely. As a result, repeated measurement is an effective way to compensate for the measurement noise.

### 5.5. Advice on Adapting Hybrid Solution

Based on all the above parametric studies, the following advice regarding the usage of the hybrid analysis is given.

(1) Measurement

A relative distance resolution less than 1 × 10^−4^ and an angular resolution less than 1 × 10^−3^ are preferable. A larger density of scanning point clouds is favored because it provides more scanning points, hence contributing to more accurate results of the hybrid analysis. Furthermore, repeated measurements are recommended.

(2) Data process

If repeated measurements are conducted, the average indicating value of every scanning point should be employed as input because it helps to reduce the measurement noise.

## 6. On-Site Application

### 6.1. Introduction of On-Site Application

**Figure 10:**
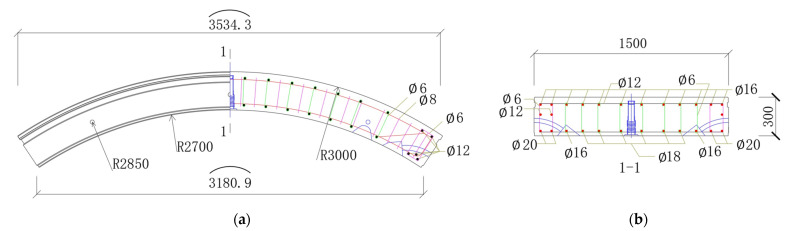
(**a**) The layout of segment B2 and (**b**) the sectional view of cross-section 1-1 (unit: mm).

**Figure 11:**
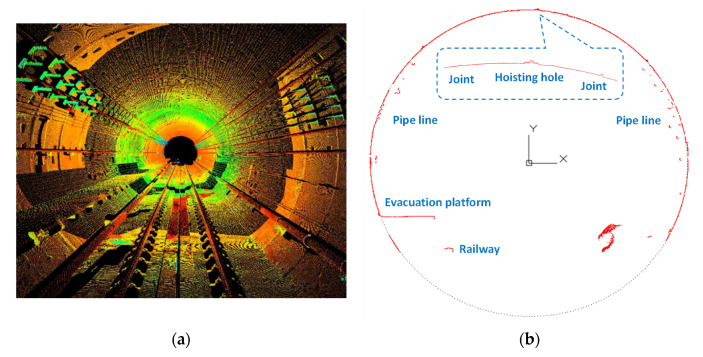
(**a**) The layout of the metro tunnel and (**b**) the corresponding laser scanning results.

To verify the feasibility of the hybrid structural analysis on actual segmental tunnel linings, laser scanning was conducted in a metro tunnel in China. The scanned lining ring has an outer diameter of 6.0 m and an inner diameter of 5.4 m. The width of the lining ring is 1.5 m and the thickness is 0.3 m. The segment materials are C50 concrete and HRB400 steel bars, where HRB400 represents hot-rolled deformed bars with a yield strength of 400 MPa. Taking segment B2 as an example, the reinforcement configuration is shown in Figure 10. The laser scanner employed on-site is the Z+F profiler 9012^®^, which has a distance resolution of 0.1 mm (corresponding to a relative distance resolution of 3.7 × 10^−5^) and an angular resolution of 1.5 × 10^−4^ rad. The noise of distance measurement is 0.5 mm and the accuracy of angular measurement is 1.22 × 10^−4^ rad. According to the parameter analysis in Section 5, the laser scanner meets the requirements of hybrid analysis. A total of 16,996 scanning points were captured, as is shown in Figure 11. It can be seen from Figure 11 that not only the surface of the segment but also the profiles of the operating system, such as the pipelines and evacuation platform, were captured with accuracy.

### 6.2. Processing of Scanned Data

**Figure 12:**
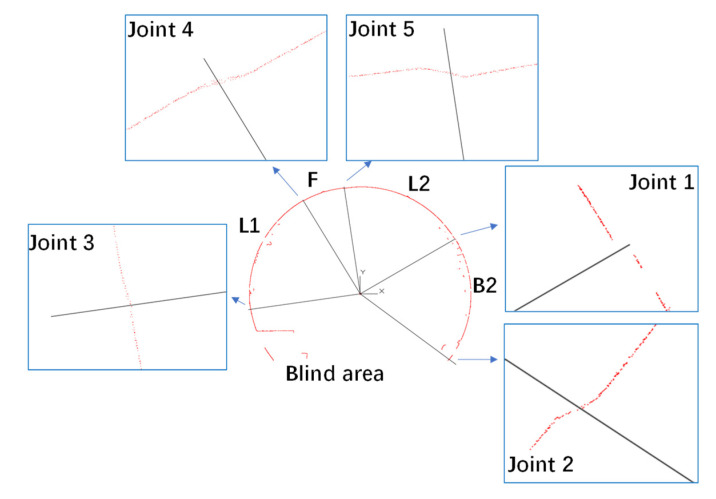
The identification of longitudinal joints.

**Figure 13:**
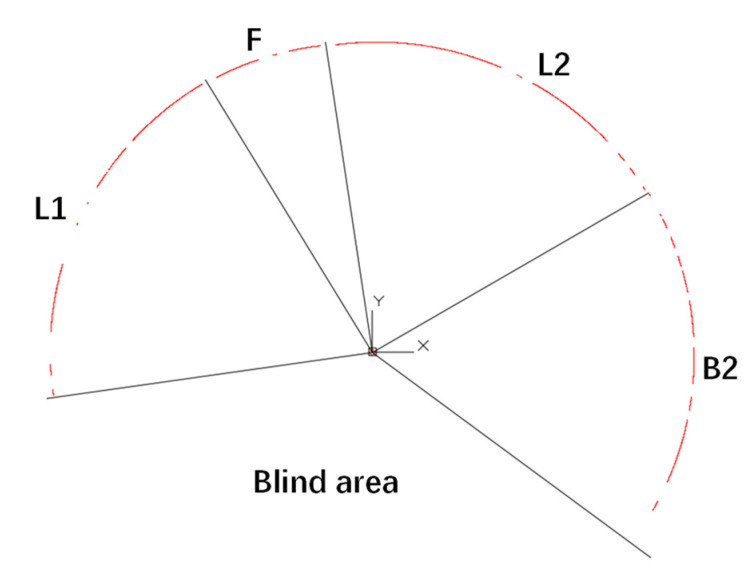
Remaining measurement points after removing equipment profile.

Because the hybrid method should be conducted segment by segment, the positions of longitudinal joints must be identified before adopting the hybrid method. The authors distinguished the joints according to the dislocation between segments, which turned out to be a feasible method; see Figure 12. Due to the existence of a track bed, the bottoms of the segment tunnel linings cannot be captured, and only four segment profiles are available, i.e., segment F, L2, B2, and L1. Then, the scanned points apart from the segment surface were recognized and removed from the database manually. There were 9613 remaining measurement points, enough for the hybrid analysis (see Figure 13).

Next, the coordinates of remaining measurement points were employed as the input of the hybrid solution, as well as the two remedial solutions. The coordinates of the inner surface of segment can represent the coordinates of the beam element because the thickness of the segment (0.3 m) is sufficiently small compared to the diameter (5.4 m inner/6.0 m outer) of the segmental lining ring. The calculation procedure of hybrid analysis follows RS1→RS2→RS3→RS1’→RS2’→RS3’→RS4’→S2→S3→S4 and the results will be discussed in Section 6.3.

### 6.3. Results and Discussion

**Figure 14:**
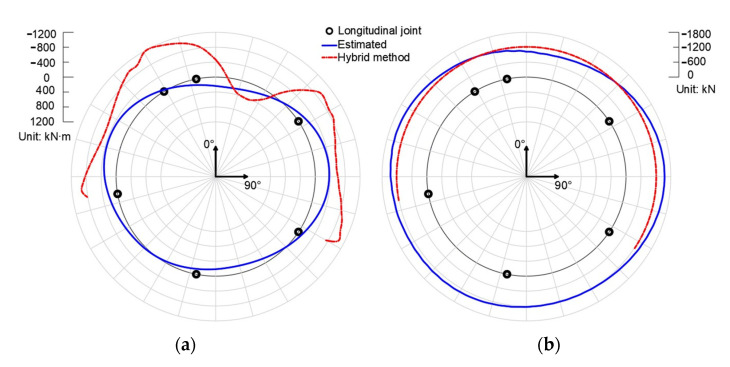
The results of bending moment (**a**) and axial force (**b**) of hybrid solution on a real segmental lining ring.

The results of the hybrid method are shown in Figure 14. The blue line represents the results of the load-controlled FEM model, where the load is obtained on-site; thus, the blue line could be regarded as well-estimated internal forces. Generally speaking, the top and bottom of the segmental lining ring are positively bended, while the two waists are in negative bending. Due to the constraints of adjacent rings, the distribution of bending moments is not strictly symmetrical about the 0–180° axis. As for the axial forces, the distribution is almost homogenous. The red line is the results of the hybrid solution. Because of the blind area caused by the track bed, the results of only four segments are presented. The bending moment distribution of the hybrid method is similar to that of the estimated one, except for a narrower positive-bending area on the top and a relatively larger quantity. The axial force of the hybrid method is estimated from the gravity of the stratum; thus, it is homogenous and in good consistency with the estimated one.

**Figure 15:**
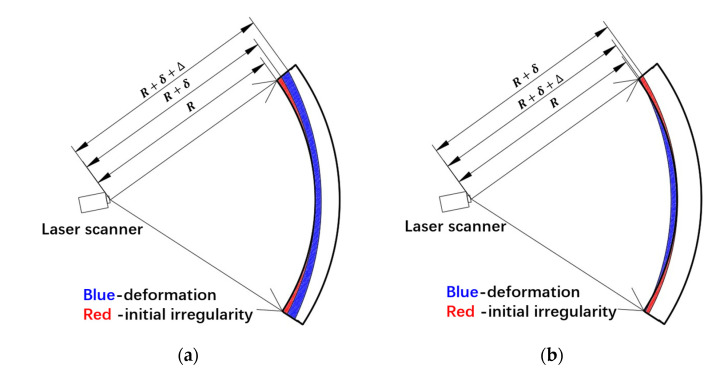
Possible initial irregularity of (**a**) segment B2, L1, and (**b**) segment L2.

The bending moment of the hybrid method is not as consistent with the estimated one as expected. Considering the strict procedure of laser scanning in the actual tunnel, the error may be caused by following reasons:

(1) Only the displacements caused by stress are considered, and the displacements caused by creep and the environment are ignored. Thus, the stress-caused displacement is overrated, which may result in a larger bending moment.
(2) In the hybrid analysis, the initial shape of the segmental tunnel lining is assumed to be a circular ring, which may differ from the real initial shape, leading to an error of bending moment calculation. As shown in Figure 14a, the bending moment distributions of segment L2, B2, and L1 are similar, where they all activate higher absolute values at two ends of the segment and lower absolute values in the middle. This may be caused by an initial irregularity shown in Figure 15, where the initial radius at the segment end is larger than the designed radius R. Because the segments are prefabricated in the same kind of modulus, every segment has identical irregularities. In Figure 15, δ is defined as the initial irregularity and Δ denotes the real deformation of the segment caused by internal forces. The initial segmental lining is assumed to be a circular ring with designed radius R; thus, the deformation Δ is misrecognized as δ+Δ in the hybrid analysis. For segment B2 and L1, they are at the negative-bending area of the segmental linings, where the deformation Δ is outward along the radial direction. Thus, the misrecognition of Δ as δ+Δ induces a larger bending moment at two ends of the segment. For segment L2, it is at the positive-bending area of the segmental linings, where the deformation Δ is inward along the radial direction. The misrecognition of Δ as δ+Δ compensates for the deformation to some extent, causing a narrower positive-bending area.

Due to the lack of historical monitoring data, the authors cannot validate the above two conjectures. They will be further discussed in future research. Nevertheless, the hybrid method is a promising method for internal force identification in the condition of limited knowledge of load, benefiting possible fault detection and safety evaluation of segmental tunnel linings.

## 7. Conclusions

This paper provides solutions regarding the combination of laser scanning and hybrid structural analysis, and the following conclusions are drawn:

(1) The original hybrid analysis is built based on the displacement-controlled finite element method, where radial displacement, tangential displacement, and rotation are employed as input. A trigonometric function is recommended by the authors to predict the displacement of unmeasured nodes. The correctness of the original hybrid solution relies on high accuracy of displacement data acquisition.
(2) The remedial solution I, designed for a lacking development process of deformed configuration, allows the backtracking of the displacement based on a deformed tunnel profile. Remedial solutions II are proposed for adopting the hybrid method at the condition of poor measurement precision. These two remedial solutions provide the feasibility to combine the hybrid method and laser scanning.
(3) Both the original hybrid method and remedial solution I and II agree well with the results of the virtual test, except for the slight deviation of the bending moment in the vicinity of longitudinal joints, which is due to the natural discontinuity of the reconstructed displacement field. Even so, the rationality of the original hybrid method and remedial solution I and II is validated.
(4) Parametric analyses were conducted to study the error propagation from laser scanning to hybrid analysis. According to the results, a relative distance resolution less than 1 × 10^−4^ and an angular resolution less than 1 × 10^−3^ are preferable for adapting the hybrid analysis on-site with laser scanning. A larger density of scanning point clouds and repeated measurements are favored, since they contribute to more accurate results of the hybrid analysis.
(5) Combination of hybrid analysis and laser scanning was conducted in a metro tunnel in China. The bending moment of the hybrid method is not as consistent with the estimated one as expected. It may be due to (1) ignoring the displacement caused by creep and the environment; (2) the imperfect manufacture of the segment. This will be further discussed in the authors’ future research.

## Figures and Tables

**Table 1:** The parameters of external load.

Parameter	Value	Note
Pv [kPa]	193.7	Vertical load
Ph1 [kPa]	123.7	Lateral load at crown
Ph2 [kPa]	190.0	Lateral load at invert
Pr [kPa]	221.1	Soil resistance
G [kN]	171.9	Self-weight of the lining (total)

**Table 2:** Groups of hybrid analyses.

Group	Method	Input	Sequence
1	Original hybrid analysis	$(u_{i},v_{i}$)	S1→S2→S3→S4
2	Hybrid analysis with remedial solution I	$(r_{i},\varphi_{i}$)	RS1→RS2→RS3→S1→S2→S3→S4
3	Hybrid analysis with remedial solution II	$\mathrm{Pv}+\mathrm{errored}\;\left({u^{\prime }}_{i},{v^{\prime }}_{i}\right)$	RS1’→RS2’→RS3’→RS4’→S2→S3→S4
4	Original hybrid analysis	$\mathrm{Errored}\;\left({u^{\prime }}_{i},{v^{\prime }}_{i}\right)$	S1→S2→S3→S4

**Figure A1:**
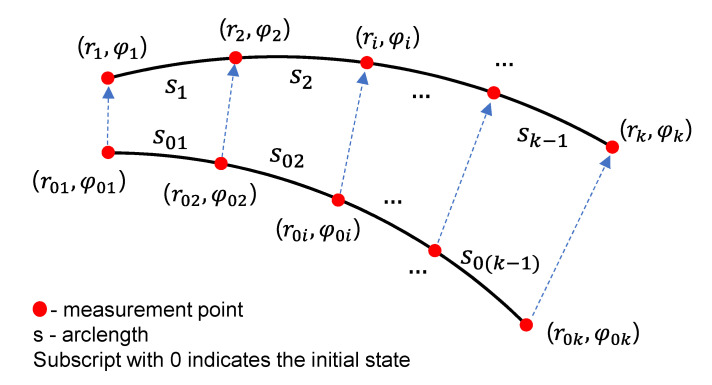
The nomenclature of Equation (A1).

## Data Availability

Not applicable.

## Appendix A. Proportionally Changing Arc Length between Measurement Points

Appendix A seeks to provide a supplementary explanation of the philosophy of remedial solution I and apply Equation (7) in Section 3.2.3. The results of the FEM model in Section 4.1 are employed and segment B2 (see Figure 3) is taken as an example. The segment is divided into 22 arcs, and the change in each arc length is extracted from the numerical model and compared. It could be seen that the change in arc length is rather small compared with the original arc length. The uniformly distributed axial forces compress the segment evenly, changing each neighboring node’s arc length to the same amount of −0.003%; see Table A1.

**Table A1:** Change in arc length of segment B2.

Number of Arcs	Original Arc Length [mm]	Deformed Arc Length [mm]	%
1	163.625	163.619	−0.003%
2	163.625	163.619	−0.003%
3	163.625	163.619	−0.003%
4	163.625	163.619	−0.003%
5	163.625	163.619	−0.003%
6	163.625	163.619	−0.003%
8	163.625	163.619	−0.003%
9	163.625	163.619	−0.003%
10	163.625	163.619	−0.003%
11	163.625	163.619	−0.003%
12	163.625	163.619	−0.003%
13	163.625	163.619	−0.003%
14	163.625	163.619	−0.003%
15	163.625	163.619	−0.003%
16	163.625	163.619	−0.003%
17	163.625	163.619	−0.003%
18	163.625	163.619	−0.003%
19	163.625	163.619	−0.003%
20	163.625	163.619	−0.003%
21	163.625	163.619	−0.003%
22	163.625	163.619	−0.003%

Therefore, it is reasonable to consider that the arc length is proportionally changing during the process of deformation:

(A1) $$\frac{s_{01}}{s_{1}}=\frac{s_{02}}{s_{2}}=\ldots =\frac{s_{0\left(k-1\right)}}{s_{k-1}},$$

The nomenclature of the equation is explained in Figure A1, where the red dots represent the measurement points and $s_{01}\sim s_{0\left(k-1\right)}$ denote the arc length at the initial state, while $s_{1}\sim s_{k}$ stand for the arc length of the deformed lining.

Based on Equation (A1), the following integration equation is built:

(A2) $$\frac{\int_{\varphi_{01}}^{\varphi_{0i}}r_{0}d\varphi }{\int_{\varphi_{01}}^{\varphi_{0k}}r_{0}d\varphi }=\frac{\int_{\varphi_{1}}^{\varphi_{i}}\sqrt{r^{2}\left(\varphi \right)+{r^{\prime }}^{2}\left(\varphi \right)}d\varphi }{\int_{\varphi_{1}}^{\varphi_{k}}\sqrt{r^{2}\left(\varphi \right)+{r^{\prime }}^{2}\left(\varphi \right)}d\varphi },$$

where $r^{\prime }\left(\varphi \right)$ is the first derivation of r(φ) in RS1’ of Section 3.2.3.$\;r_{0}$ is the design radius of the lining ring, which is a constant. $\varphi_{0i}$ and $\varphi_{i}$ are the initial and current polar angle of the measurement point i, respectively, as is shown in Figure A1. The subscripts 1 and k denote the number of measurement points at the two ends of a segment, respectively. Rearranging Equation (A2), the initial polar angle of measurement point i reads as Equation (A3), which is the same as Equation (8) in Section 3.2.3:

(A3) $$\varphi_{0i}=\frac{\int_{\varphi_{1}}^{\varphi_{i}}\sqrt{r\left(\varphi \right)+r^{\prime }\left(\varphi \right)}d\varphi }{\int_{\varphi_{1}}^{\varphi_{k}}\sqrt{r\left(\varphi \right)+r^{\prime }\left(\varphi \right)}d\varphi }\left(\varphi_{0k}-\varphi_{01}\right)+\varphi_{01},$$

## Appendix B. List of Symbols

**Table:**

Abbr.	Meaning	Abbr.	Meaning
A1~A5	Assumptions of hybrid analysis	S1~S4	Steps of original hybrid analysis
A	Cross-sectional area of segment	u	Displacement vector
b	Width of segment	$u_{e}$	Displacement vector of element node
$C_{1}\sim C_{5}$	Undetermined constants for u interpolation function	u	Radial component of u
${C^{\prime }}_{1}\sim {C^{\prime }}_{5}$	Undetermined constants for v interpolation function	$u_{ei}$	Radial component of $u_{e}$
${C^{\prime \prime }}_{1}\sim {C^{\prime \prime }}_{5}$	Undetermined constants for r interpolation function	$u_{i}$	Radial displacement of measurement point
E	Elastic modulus of concrete	${u^{\prime }}_{i}$	Errored radial displacement of measurement point
G	Self-weight of the lining	v	Tangential component of u
$H_{i}$	Height of each stratum layer	$v_{ei}$	Tangential component of $\;u_{e}$
N	Axial force of lining ring	$v_{i}$	Tangential displacement of measurement point
Ph1	Lateral load at crown	${v^{\prime }}_{i}$	Errored tangential displacement of measurement point
Ph2	Lateral load at invert	$X_{1}\sim X_{3}$	Undetermined constants for v interpolation function
Pr	Soil resistance	$\gamma_{i}$	Gravity density of stratum layer
Pv	Vertical load	θ	Cross-sectional rotation
r	Configuration function of segment	$\theta_{ei}$	Cross-sectional rotation of element node
r	Current radial coordinate of segment	$\theta_{i}$	Cross-sectional rotation of measurement point
$r_{0i}$	Initial radial coordinate of measurement point	σ	Distance noise of laser scanning
$r_{i}$	Current radial coordinate of measurement point	$\sigma_{r}$	Relative distance noise of laser scanning
R	Average radius of the lining ring.	φ	Angular coordinate of the polar coordinate system
Res	Distance resolution of laser scanning	$\varphi_{i}^{e}$	Angular coordinate of element node
$Res_{r}$	Relative distance resolution of laser scanning	$\varphi_{0i}$	Initial angular coordinate of measurement point
RS1–RS3	Steps of remedial solution I	$\varphi_{i}$	Current angular coordinate of measurement point
RS1’–RS4’	Steps of remedial solution II		
